# Professional Appointments

**Published:** 1860-10

**Authors:** 


*Professional Appointments.*—Dr. E. Warren, of N. C., formerly editor of the N. C. Journal of Medicine, has received the appointment to the Chair of Materia Medica in the University of Maryland, vacated by the death of Dr. C. Frick. Dr. W. A. Hammond, U. S. A., has been appointed to the Chair of Anatomy and Physiology in the same school to fill the resignation of Prof. Roby. B.

